# Highly Sensitive Duplex Quantitative PCR Assay for Simultaneous Detection of Two Japanese Eel Viruses, Anguillid Herpesvirus 1 and Japanese Eel Endothelial Cells-Infecting Virus

**DOI:** 10.3390/biology14030264

**Published:** 2025-03-05

**Authors:** Jun-Young Song, Keun-Yong Kim, Ahran Kim

**Affiliations:** 1 Department of Aquatic Life Medical Sciences, Sunmoon University, Asan 31460, Republic of Korea; jysong80@sunmoon.ac.kr; 2 Genetic Analysis Team, AquaGenTech Co., Ltd., Busan 48228, Republic of Korea; aquagentech@naver.com; 3 Pathology Division, National Institute of Fisheries Science, Busan 46083, Republic of Korea

**Keywords:** AnHV, aquaculture, diagnostics, eels, JEECV, qPCR

## Abstract

Japanese eel endothelial cells-infecting virus (JEECV) and Anguillid herpesvirus 1 (AnHV) are major pathogens in farmed eels. JEECV causes eel viral endothelial cell necrosis (VECNE), while AnHV leads to symptoms such as head erythema and gill necrosis. Both viruses cause severe mortality alone or in combination, necessitating rapid and early detection of their presences. In this study, we developed a highly efficient duplex quantitative PCR method (*r*^2^ = 0.999) using hydrolysis probes for the rapid and simultaneous detections of AnHV and JEECV. This new diagnostic method demonstrated a 1.7-fold higher detection rate for AnHV and a 2.5-fold higher detection rate for JEECV than conventional PCR. Quantitative analysis of water and eel tissue samples from aquaculture facilities revealed that the two viruses could be detected in water 1–3 months prior to mortality, enabling their early identification of infections through water testing alone. Notably, the method reliably detected low viral loads (< 1 copy) in both water and tissue samples, facilitating preclinical detection and proactive disease management. This approach reduces the risk of mass mortality and economic losses in eel farming. This study underscores the critical role of advanced molecular diagnostic technologies in enhancing health management in aquaculture.

## Simple Summary

Farmed eel production is severely threatened by two viruses—the Japanese eel endothelial cells-infecting virus and Anguillid herpesvirus 1. In the event of an outbreak of these two viruses, the recirculating aquaculture systems used in eel farms facilitate rapid disease transmission across tanks. This not only leads to mass mortality but also to substantial economic losses. Thus, their accurate early detection is required to ensure rapid responses and mitigate this risk. In this study, we developed a quantitative PCR method capable of simultaneously detecting both viruses, and it was superior to conventional PCR methods. Water and eel tissue samples from aquaculture facilities were assessed by our proposed PCR, and the results showed that the viruses could be found in the water 1 to 3 months before the eels showed signs of illness. Therefore, their infections could be detected early just by testing the water. Additionally, the method was able to consistently detect very small amounts of the viruses in both water and tissue samples. Our approach aids in the early identification of infections and management of viral diseases.

## 1. Introduction

Eels are among the most prominent species in the inland aquaculture industry of South Korea. Between 2012 and 2021, eel production increased by approximately 3.6-fold, reaching 15,764 tons in 2021, accounting for approximately 47% of the total inland aquaculture output [1]. In South Korea, most eels are reared in recirculating aquaculture systems (RASs), where water temperatures are maintained between 27 and 32 °C throughout the year, even during winter [2]. Although this stable environment optimizes eel growth, it poses significant risks. Specifically, in the event of a disease outbreak, the shared filtration systems in RASs can facilitate rapid disease transmission across tanks, potentially leading to mass mortality and substantial economic losses. Therefore, early detection and rapid response to disease outbreaks are critical to mitigating these risks.

Recent studies have reported mass mortality in cultured eel species in South Korea, including *Anguilla japonica*, *A. bicolor*, and *A. marmorata*, due to infections by two DNA viruses: Anguillid herpesvirus 1 (AnHV) and Japanese eel endothelial cells-infecting virus (JEECV) [3,4,5,6]. In eel farms, disease outbreaks caused by these viruses typically occur one month after stocking, leading to approximately 50% mortality within three months. In particular, their co-infection has been reported to result in mortality rates as high as 90%, highlighting the severe economic losses experienced by eel farms [3,4,5,6]. JEECV, the causative agent of viral endothelial cell necrosis of eel (VECNE), is characterized by symptoms such as head reddening, pectoral fin necrosis, and gill congestion [5,7,8]. AnHV, first isolated in *A. japonica* and *A. anguilla* in Japan in 1985 [9], is associated with disease outbreaks in farmed eels in Japan, Taiwan, and South Korea, and typically causes symptoms such as snout reddening, skin hemorrhages, and necrosis of the skin and gills [3,4,6,10,11,12,13]. Although they can infect eels independently, AnHV and JEECV often occur in conjunction with bacterial co-infections. They can also present as dual infections, resulting in more severe diseases and elevated mortality rates [3]. Notably, a recent survey of eel farms detected JEECV in 49% of cases, AnHV in 27%, and co-infections in 17%, highlighting their critical prevalence in aquaculture [3]. Until now, conventional PCR (cPCR) methods have been used to diagnose the two viruses separately [8,14]. However, this approach is time-consuming and thus is not practical for large-scale sample testing or urgent disease diagnosis. To address this challenge, a more rapid and high-throughput diagnostic method must be developed to improve disease management in eel farming. Moreover, developing highly sensitive diagnostic tools is crucial for enabling timely identification and response to infections.

In the present study, we aimed to establish a duplex quantitative PCR (qPCR) method utilizing hydrolysis probes for the simultaneous detection of AnHV and JEECV. We validated the efficacy of this diagnostic approach through quantitative analyses of viral presences in water and eel tissue samples from aquaculture farms. This study demonstrates the potential of this approach for early detection and improved disease control in eel farming operations.

## 2. Materials and Methods

### 2.1. Fish Sampling

Twenty-eight eels exhibiting symptoms of viral infection were collected from aquaculture farms. Viral DNA was extracted from pooled spleen and kidney samples using the DNA Isolation Kit for Cells and Tissues (Roche, Basel, Switzerland), following the manufacturer’s instructions. DNA concentration and purity were assessed with a NanoVue™ Plus Spectrophotometer (GE Healthcare, Chicago, IL, USA). The DNA was diluted to 20 ng/μL, stored at −20 °C, and used for sequencing target genes and verifying the qPCR markers developed in this study.

### 2.2. Sequencing of Target Genes

Analysis of the GenBank database revealed that the DNA polymerase catalytic subunit gene (*Pol*) of AnHV and the polyomavirus large T-antigen-like protein gene (*Ltlg*)of JEECV had the most abundant sequence information. Using these regions from AnHV (GenBank accession number: KX027736) and JEECV (NC_015123) as reference sequences, new PCR primers were designed (Table 1). PCR amplification was performed using these primers on viral DNA from the 28 eel specimens. PCR-positive samples were sequenced, and their *Pol* and *Ltlg* sequences were analyzed. The newly sequenced genes were deposited in GenBank (accession numbers: PV131738–131754) and aligned with corresponding sequences registered in GenBank. Molecular phylogenetic trees were constructed using the neighbor-joining method to establish phylogenetic relationships.

### 2.3. Design and Verification of qPCR Markers

The DNA sequences of AnHV registered in GenBank and the newly sequenced 13 AnHV-positive samples were aligned to identify a conserved region present in both AnHV-1 and AnHV-2. This conserved sequence was used to design highly specific qPCR primers and hydrolysis probes. The AnHV probe employed Cy5 as the fluorescent reporter dye and BHQ2 as the quencher (Table 2). The optimal annealing/extension temperature for the AnHV qPCR was determined by testing temperatures in 2 °C increments between 60 °C and 70 °C to identify the condition that yielded the highest amplification efficiency.

Similarly, the DNA sequences of JEECV in GenBank and the newly four JEECV-positive samples were aligned to identify a conserved region. Specific qPCR primers and hydrolysis probes were designed for JEECV detection, using HEX as the fluorescent reporter dye and BHQ1 as the quencher (Table 2). The annealing/extension temperature for JEECV qPCR amplification was optimized using the same temperature range (60–70 °C) to achieve maximum amplification efficiency.

### 2.4. Optimization of qPCR Conditions

To optimize the duplex qPCR conditions for detecting AnHV and JEECV, primer and hydrolysis probe concentrations were tested in the range of 5–10 pmol/rxn. The annealing/extension temperatures were set at 62 and 63 °C for the final verification. Detailed information on the reaction mixture and amplification conditions is provided in Table 3 and Table 4.

### 2.5. Standard Curves

Internal standards were established for the target genes (*Pol* for AnHV and *Ltlg* for JEECV). DNA fragments containing the forward and reverse primers, as well as hydrolysis probe binding sites, were synthesized and inserted into plasmids for mass production (Bioneer, Daejeon, South Korea). The plasmid DNA was quantified, and copy numbers were standardized to 100,000. Serial 10-fold dilutions were performed to establish standard materials with concentrations ranging from 100,000 copies/rxnto 1 copy/rxn. Calibration curves were generated for each virus to enable precise quantification of the target genes.

### 2.6. Sensitivity Tests

To evaluate the detection sensitivity of the diagnostic method developed in this study compared to cPCR, both cPCR and qPCR analyses were performed on 28 eel tissue samples, along with two no-template controls. All DNA samples were diluted to a concentration of 20 μg/μL, and 2 μL (40 μg) of each sample was used for the analyses. For cPCR, the primers newly designed in this study for detecting AnHV or JEECV were used (Table 1). For JEECV detection, as the virus was not identified in the first round of PCR, nested PCR was performed to enhance sensitivity.

### 2.7. Spike Tests Using Culture Water

To simulate virus detection in aquaculture water, JEECV-infected eel tissues (kidney and spleen pool) were diluted 10-fold in Minimum essential medium (MEM) and completely homogenized. Subsequently, 350 μL (containing 35 mg of tissue) of eel tissue homogenate was added to 20 L of groundwater used in eel farming and mixed thoroughly. The spiked groundwater was divided into portions of 1, 2, 4, and 10 L and filtered through cellulose nitrate membranes (50 mm diameter, 0.45 μm pore size; Whatman^®^, Maidstone, UK). Viral DNA was then extracted using the DNeasy Blood & Tissue Kit (QIAGEN, Hilden, Germany), following the complete destruction of the membranes. This destruction was achieved by adding three stainless steel beads (2.4 mm diameter; OMNI International, Kennesaw, GA, USA) and processing the sample with a Bead Ruptor Elite homogenizer (OMNI International).

### 2.8. Validation of the qPCR Diagnostic Method for Monitoring Viral Outbreaks in Eel Farms

The qPCR diagnostic method developed in this study was validated by monitoring virus occurrences in eel tissues and culture water from two eel farms monthly. One liter of rearing water was collected from aquaculture tanks and transported to the laboratory. Suspended solids were removed, and the water was filtered through a nitrocellulose membrane filter (0.45 μm pore size). Subsequently, environmental DNA (eDNA) was extracted from the membrane filter using the DNeasy Blood & Tissue Kit (QIAGEN). To monitor virus presences in eel tissues, three eels were sampled from the same tanks where water was collected. Tissues were collected from the pectoral fins, gills, kidneys, and spleen. Genomic DNA was extracted from each tissue using the DNA Isolation Kit for Cells and Tissues (Roche) and eluted in 50 μL of TE buffer (10 mM Tris-HCl, pH 8.0; 1 mM EDTA, pH 8.0). Subsequently, DNA concentration and purity were determined using a spectrophotometer. The extracted eDNA from water samples and genomic DNA from tissue samples were used as templates for quantifying viral gene copy numbers using the qPCR method

## 3. Results

### 3.1. Phylogenetic Relationships of Target Genes

**Figure 1:**
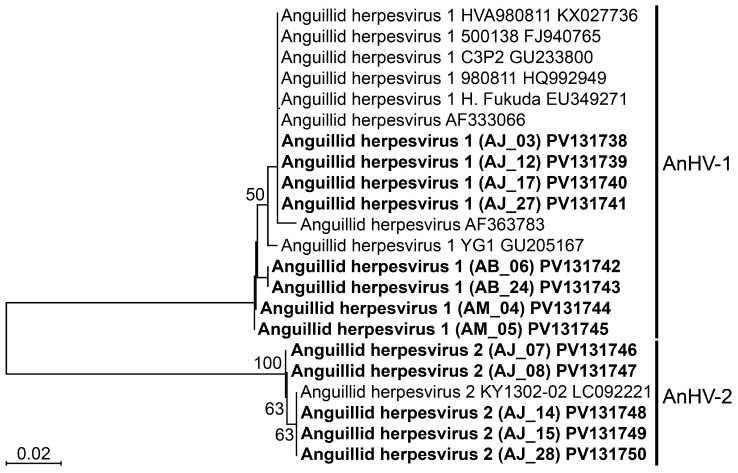
Phylogenetic tree constructed by neighbor-joining method based on the *Pol* sequences of AnHV. The sequences analyzed in this study were in the bold.

Primers designed for AnHV (Table 1) were utilized for nested PCR amplification, confirming its presence in 13 out of 28 eel samples. Sequencing analysis of *Pol* from the amplified products revealed that all AnHV-positive samples were successfully sequenced, with total lengths ranging from 825 to 846 bp. Their length variations were attributed to the insertion of four codons and the deletion of three codons in *Pol* compared to the reference sequence from GenBank database (accession number: KX027736). A phylogenetic tree constructed using the neighbor-joining method (Figure 1) revealed two distinct genotypes, type 1 and type 2, within AnHV. Notably, all AnHV-positive samples with the type 2 genotype exhibited no codon deletions.

**Figure 2:**
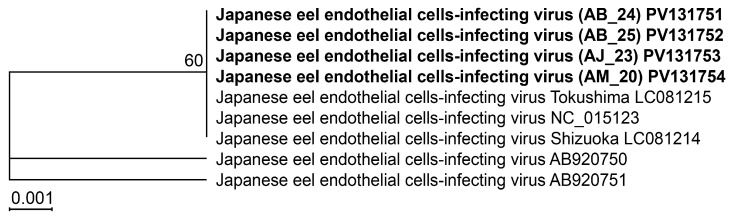
Phylogenetic tree constructed by neighbor-joining method based on the *Ltlg* sequences of JEECV. The sequence analyzed in this study were bold.

Similarly, primers designed for JEECV (Table 1) were used for nested PCR amplification, identifying four JEECV-positive samples. Sequencing analysis of *Ltlg* from the amplified products revealed the uniform sequence length of 723 bp across all samples, with 100% identity and no observed genetic variation. These sequences were aligned with the reference sequence (accession number: NC_015123), and a phylogenetic tree was subsequently constructed using the neighbor-joining method (Figure 2). The analysis confirmed the absence of genetic diversity among the JEECV-positive samples in this study.

### 3.2. Optimization of Duplex qPCR

To optimize duplex qPCR conditions for detecting AnHV and JEECV, the newly designed primers and hydrolysis probes (Table 2) were tested. Primer and probe concentrations were evaluated in the range of 5–10 pmol/rxn, and annealing/extension temperatures were tested at 62 and 63 °C. At 62 °C, weak nonspecific signals were observed in the negative control, whereas no nonspecific signals occurred at 63 °C. These nonspecific signals were enhanced with increasing primer and probe concentrations; therefore, the optimal concentration was determined to be 5 pmol/rxn. Consequently, the optimal duplex qPCR conditions were set at an annealing/extension temperature of 63 °C with primer and probe concentrations of 5 pmol/rxn.

### 3.3. Standard Curve

**Figure 3:**
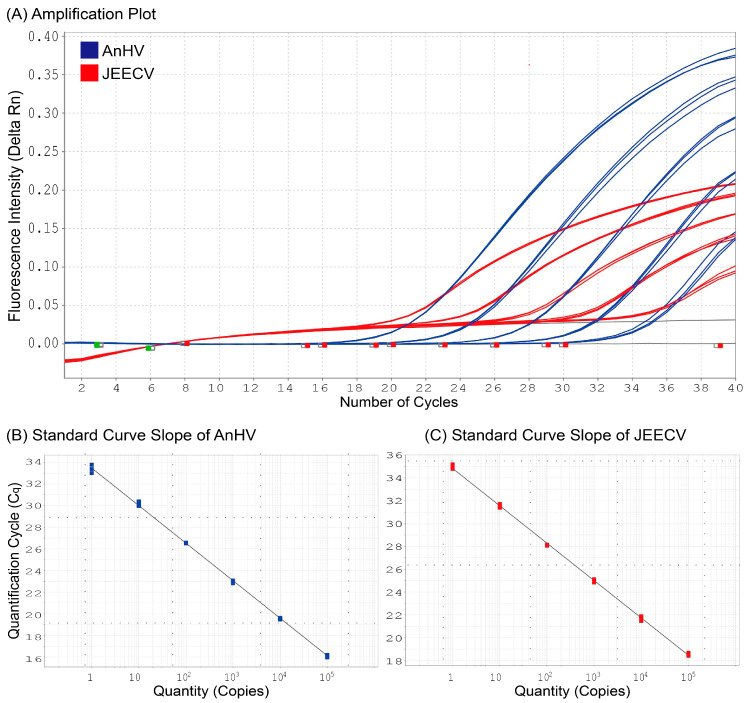
Duplex qPCR amplification curves of AnHV and JEECV (**A**) and standard curves for AnHV (**B**) and JEECV (**C**) at internal standard concentrations (100,000 copies/rxn, 10,000 copies/rxn, 1000 copies/rxn, 100 copies/rxn, 10 copies/rxn, 1 copy/rxn), with an *r*^2^ value of 0.999.

To establish internal standards for the target gene regions—*Pol* for AnHV and *Ltlg* for JEECV—PCR amplification products were cloned into plasmid DNA using TA cloning for large-scale replication. The plasmid DNA was standardized to 100,000 copies based on molecular weight and concentration. Serial 10-fold dilutions were then prepared to generate standard materials with concentrations ranging from 100,000 copies/rxn to 1 copy/rxn. Standard curves were constructed for each virus, demonstrating a strong inverse relationship between C_q_ values and standard material concentration, with an *r*^2^ value of 0.999, indicating high reliability. Notably, nonspecific signals were not detected in the negative control (Figure 3).

### 3.4. Sensitivity of the qPCR Marker

The sensitivity of the duplex qPCR method, developed to detect AnHV and JEECV, was compared using cPCR on 28 eel samples (Table 5). For AnHV, cPCR identified positive bands in 7 out of 28 samples. In comparison, the duplex qPCR method detected 12 positive samples, representing a 1.7-fold increase in sensitivity. All cPCR-positive samples were also identified as positive using qPCR, with viral loads ranging from 167.6 to 76,209.1 copies. Notably, samples that were identified as negative using cPCR but positive using qPCR had viral loads ranging from 0.2 to 218 copies. For JEECV, cPCR failed to detect any positive samples in the initial PCR step, and only four samples were confirmed positive using nested PCR. In contrast, the duplex qPCR method identified 10 positive samples, achieving a 2.5-fold higher detection rate than that of nested PCR. Viral loads in qPCR-positive samples ranged from 0.1 to 2104 copies.

### 3.5. Spike Tests on Culture Water

JEECV was successfully detected in groundwater samples (1, 2, 4, and 10 L) spiked with 4 mg/L of JEECV-infected tissue homogenate. The viral load increased proportionally with the volume of concentrated groundwater (Table 6). Specifically, the viral load in 2 L of concentrated groundwater was approximately 3.6 times higher (44,250 copies) than that in 1 L (12,367 copies). It also increased by 14-fold (173,072 copies) and nearly 30-fold (375,871 copies) in samples with volumes of 4 and 10 L, respectively. In contrast, no virus was detected in the corresponding negative control groundwater samples, regardless of volume.

### 3.6. Validation of the qPCR Diagnostic Method for Monitoring Viral Outbreaks in Eel Farms

Quantitative analysis of viral DNA from eDNA and eel tissue samples (gills, fins, kidneys, and spleen) collected from two eel farms is summarized in Table 7. In Farm A, no viruses were detected in either the eDNA or eel tissues during the first survey. During the second survey, JEECV was detected in the eDNA at a concentration of 1.0 × 10^1^ copies/mL. By the third survey, JEECV was detected in all eDNA and eel tissues, with the highest concentration observed in the gills at 4.0 × 10^3^ copies/μL. Mortalities were also reported during this survey. In Farm B, AnHV was detected in the eDNA during the first survey; however, its concentration was below the quantification limit (< 1 copy/mL), preventing precise quantification. In the second survey, no virus was detected in the eDNA, whereas AnHV was identified in all eel tissues, particularly in the fins and gills of all three sampled eels, with additional detections in some kidney and spleen samples. During the third survey, no virus was detected, although the eels appeared weak. In the fourth survey, both eDNA and eel tissue samples tested positive for the virus, and mortality events were recorded.

## 4. Discussion

In a previous study, we investigated the infection status of AnHV and JEECV in eel farms across South Korea [3], confirming that they are widespread in cultured eels, with frequent cases of co-infection. Such mixed infections, particularly during the juvenile stage, often led to mass mortality, resulting in significant economic losses. These findings emphasize the need for monitoring both viruses to improve disease surveillance in eel farms. However, the currently available PCR methods [8,14] require individual detection of each virus, making the process labor-intensive and time-consuming. To address these limitations, in the present study, we developed a highly sensitive duplex qPCR method capable of detecting both viruses simultaneously.

We conducted DNA sequencing of PCR-positive samples after designing primers specific genes of *Pol* and *Ltlg* to JEECV and AnHV, respectively. While the sequencing results revealed no genetic variation in JEECV, two types of AnHV (AnHV-1 and AnHV-2) were detected, independent of host species or geographic region. AnHV-1 has been documented in various eel species, including *A. anguilla*, *A. japonica*, *A. bicolor*, *A. marmorata*, and *A. rostrata*, across Europe, East Asia, and Southeast Asia [6,15,16,17,18,19], with full genome sequences reported for strains from *A. anguilla* and *A. rostrata* [20,21]. AnHV-2, first isolated from *A. japonica*, has been partially sequenced for *Pol* (GenBank accession number: LC092221) serving as a reference. While Kim et al. have recently developed a duplex PCR method for detecting AnHV-1 [22], in this study, we designed primers and probes targeting conserved regions of the *Pol* gene shared by both AnHV-1 and AnHV-2. This approach enabled us to simultaneously detect both AnHV types. Moreover, standard curve analysis using plasmid DNA demonstrated high reliability (*r*^2^ = 0.999) in detecting both viruses down to a single copy, confirming the exceptional sensitivity of our method for detecting trace viral loads. In addition, compared to cPCR, the duplex qPCR method developed in this study was 1.7 times more sensitive for AnHV and 2.5 times more sensitive for JEECV. The ability of our method to detect low viral loads undetectable by cPCR significantly enhances the accuracy of disease monitoring. This heightened sensitivity enables the detection of viral infections at preclinical stages, prior to the onset of symptoms, thereby facilitating early diagnosis and intervention for AnHV and JEECV in farmed eels.

In addition, using this diagnostic tool, we conducted a six-month monthly monitoring program at two eel farms and analyzed samples from rearing water and fish. We observed no cases of co-infection during the monitoring period; however, we detected AnHV and JEECV individually. At Farm A, initial investigations revealed no detectable viral DNA in either eel tissues or eDNA, suggesting either pre-infection conditions or a viral load too low to detect. In subsequent investigations, JEECV was detected in eDNA at a concentration of 1.0 × 10^1^ copies/mL, indicating initial viral introduction. By the third investigation, JEECV was detected in all eel tissues, coinciding with an increase in eel mortality. The highest viral concentration (4.0 × 10^3^ copies/μL) was observed in the gills, suggesting that this tissue is the primary entry point and reservoir for infection. At Farm B, AnHV was initially detected in eDNA from rearing water at concentrations below the limit of quantification (< 1 copy/mL), indicative of latent or low-level infection. In the second survey, the virus was undetectable in eDNA; however, it was present in all eel tissues, highlighting the importance of tissue sampling for identifying subclinical infections that may be missed by eDNA analysis alone. During the third survey, the virus was not detected, despite deterioration in eel health. By the fourth survey, AnHV was detected in all samples, and mortality rates had increased, marking the onset of an outbreak. These findings emphasize the necessity of continuous monitoring and early intervention to prevent mass mortality from viral infections.

The present study suggests that an integrated sampling approach, combining rearing water eDNA analysis with fish tissue examination, may provide a more accurate prediction of viral disease outbreaks than an individual approach. However, repeated sampling of live fish can impose significant stress on the stock. Therefore, continuous monitoring of rearing water eDNA using the high-sensitivity detection technology developed in this study offers an efficient and non-invasive alternative for viral disease surveillance and control. Moreover, when the timing of virus detection in stock water was compared to the onset of eel mortality, viruses were consistently detected in eDNA from three weeks to three months before mortalities were observed (Table 7). This finding demonstrates that monitoring eDNA in rearing water alone is sufficient to predict disease outbreaks. Similarly, recent studies have highlighted the potential of eDNA methods for detecting fish viruses [23,24,25,26]. Notably, DNA viruses are more stable than RNA ones, making them particularly suitable for eDNA-based monitoring [27]. Given that they are DNA viruses, both AnHV and JEECV serve as ideal candidates for detection using this method in aquaculture settings. Moreover, the highly sensitive eDNA detection technology developed in this study can be applied to the surveillance of other aquatic pathogens in various aquaculture systems and natural waters as well as in eel farms. This approach may be useful for the early detection and control of various diseases affecting fish, thereby contributing to improving aquatic animal health management on a broader scale.

This study has some limitations that should be considered. First, the data were collected from only two eel farms, which may not fully represent the situation across all aquaculture farms. Expanding the sample size in future research would improve the generalizability of the findings. Second, environmental factors such as water temperature, quality, and stocking density, which are known to influence viral outbreaks, were not included in the analysis. Future studies incorporating these factors could provide more comprehensive insights into disease dynamics. Third, while we used filtration methods to detect viruses in rearing water, further comparative studies exploring alternative concentration methods, such as ultracentrifugation, may enhance the detection efficiency and reliability of viral monitoring techniques. Lastly, while the eDNA method showed high sensitivity, further investigation is needed to clarify the relationship between eDNA concentration and actual infection status, as well as the stability and degradation of eDNA under various environmental conditions. Nevertheless, this study offers essential baseline data that will contribute to the development of effective virus monitoring and early warning systems for aquaculture farms.

## 5. Conclusions

In conclusion, we developed a highly sensitive diagnostic technology capable of simultaneously detecting both AnHV and JEECV, which are major causes of mortality in eel farms. The method is specifically designed to detect two types of AnHV. With the sensitivity to detect as little as a single plasmid DNA copy, this technology supports non-destructive testing using fin or gill samples, obviating the need to dissect internal organs. Furthermore, we confirmed that virus monitoring is feasible using eDNA from rearing water. Notably, eDNA-based virus monitoring eliminates the need for complex and invasive fish sampling procedures, enabling early detection of infections and prompt intervention to prevent disease spread. Our results also indicate that virus detection can be achieved with only 1 L of culture water, although elevated concentrations of culture water enhance detection sensitivity. These findings highlight eDNA-based monitoring as a practical tool for early diagnosis and outbreak prediction. This diagnostic technology has broad applications in viral disease monitoring, vaccine development, and treatment research, offering a significant contribution to advancing health management practices in eel farming.

## Figures and Tables

**Table 1:** Primer sequences designed for sequencing of target genes of AnHV and JEECV.

Virus	Target Gene	PCR	Primer Name	Sequence (5′ → 3′)	Amplicon Size (bp)	Reference
AnHV	*Pol*	1st	2061f	CAAGCTGAAGGAGAACAGAT	1045–1054	This study
			3086r	GTTCGTGATCAGAGAGTTGT		This study
		Nested	2108f	CCATGTGCTTGACTGAAAAG	825–846	This study
			3016r	TCCTCAAAGAACCACGCTTT		This study
JEECV	*Ltlg*	1st	0343f	TTTGTGCAGACTGTACTGGT	1033	This study
			1455r	TACTCATTCATAGTGGCAATC		Modified from Mizutani et al. [8]
		Nested	0462f	AACAGGCATAGCAGTTGTGC	723	This study
			1219r	TCACCGTTCATGTTTAGGAC		Modified from Mizutani et al. [8]

**Table 2:** Primer and probe sequences designed for duplex qPCR of AnHV and JEECV.

Target Virus	Primers and Probe	Sequence (5′–3′)
AnHV	AnHV-Pol-2173f	AAGGTGTTTCAGCCYACCAT
	AnHV-Pol-2587r	ATGAAGATCT GCGCCAACTC
	AnHV-Pol-2260p	Cy5-AGCAACATGTGCGACGCCAA-BHQ2
JEECV	JEECV-Ltlg-0546f	CAATGTGATGCAGGTAGCAA
	JEECV-Ltlg-0777r	TCTGTTGGTCGCTTCGACAT
	JEECV-Ltlg-0662p	HEX-TGGGCTTTGACTACACGATGCT-BHQ1

**Table 3:** Reagents used for duplex qPCR of AnHV and JEECV.

Reagents	Amount
2× qPCR Master Mix	10 μL
Forward Primer (5–10 pmol)	1–2 μL
Reverse Primer (5–10 pmol)	1–2 μL
TaqMan Probe (5–10 pmol)	1–2 μL
ROX™ dye	0.1 μL
Template DNA	2 μL
DEPC-treated Water	up to 20 μL
Total	20 μL

**Table 4:** Optimized duplex qPCR conditions of AnHV and JEECV in this study.

Step	Temperature	Time	No. of Cycles
Initial denaturation	95 °C	5 min	1
Denaturation	95 °C	15 s	40
Annealing/Extension	63 °C	1 min	

**Table 5:** Comparison of the diagnostic performance of the proposed qPCR method and cPCR using eel tissues.

No.	Host	Area	Fish Size (cm)	AnHV		JEECV	
				cPCR	qPCR (Copies/mL)	cPCR ^2^	qPCR (Copies/mL)
1	*A. japonica*	Jeollabuk-do	50.5	ND ^1^	ND	ND	ND
2	*A. japonica*	Jeollanam-do	48.2	ND	ND	ND	1.1
3	*A. japonica*	Jeollanam-do	42.3	Positive	2308.9	ND	2.9
4	*A. marmorata*	Gyeongsangbuk-do	50.9	ND	ND	ND	ND
5	*A. marmorata*	Gyeongsangbuk-do	11.6	Positive	76,209.1	ND	ND
6	*A. bicolor*	Gyeongsangnam-do	No data	Positive	711.7	ND	0.1
7	*A. japonica*	Jeollabuk-do	No data	ND	ND	ND	2104.5
8	*A. japonica*	Jeollabuk-do	56.7	ND	11	ND	0.8
9	*A. australis*	Jeollanam-do	14.5	ND	ND	ND	ND
10	*A. australis*	Jeollanam-do	12.6	ND	ND	ND	ND
11	*A. australis*	Jeollanam-do	55.8	ND	ND	ND	ND
12	*A. japonica*	Chungcheongnam-do	53	Positive	1668.7	ND	ND
13	*A. japonica*	Jeollanam-do	24.6	ND	0.6	ND	0.5
14	*A. japonica*	Jeollanam-do	47	ND	28	ND	ND
15	*A. japonica*	Jeollanam-do	43.5	ND	0.3	ND	ND
16	*A. bicolor*	Gyeongsangnam-do	39.5	ND	ND	ND	ND
17	*A. japonica*	Jeollanam-do	46.5	Positive	167.6	ND	ND
18	*A. australis*	Jeollanam-do	13.9	ND	0.2	ND	ND
19	*A. japonica*	Jeollanam-do	59.5	ND	ND	ND	ND
20	*A. marmorata*	Gyeongsangbuk-do	52.8	ND	ND	Positive	ND
21	*A. bicolor*	Gyeongsangnam-do	42.2	ND	ND	ND	ND
22	*A. australis*	Jeollanam-do	48	ND	ND	ND	ND
23	*A. japonica*	Jeollabuk-do	53.4	ND	ND	Positive	2.5
24	*A. bicolor*	Gyeongsangnam-do	No data	Positive	464.9	Positive	0.3
25	*A. japonica*	Jeollabuk-do	No data	ND	ND	Positive	1182.9
26	*A. australis*	Jeollanam-do	55.8	ND	ND	ND	ND
27	*A. japonica*	Chungcheongnam-do	53	Positive	218.5	ND	ND
28	*A. japonica*	Jeollanam-do	24.6	ND	0.2	ND	0.3
29	NTC-1	-		ND	ND	ND	ND
30	NTC-2	-		ND	ND	ND	ND

^1^ ND, not detected; ^2^ Nested PCR result of JEECV.

**Table 6:** Results of the spike test using JEECV-positive eel tissue homogenate in groundwater.

Sample Name	Sample Volume (L)	AnHV		JEECV	
		C_q_ Value	Copy No.	C_q_ Value	Copy No.
Groundwater	1	ND	-	ND	-
Groundwater	2	ND	-	ND	-
Groundwater	4	ND	-	ND	-
Groundwater	10	ND	-	ND	-
Spiked groundwater	1	ND	-	21.424	12,367
Spiked groundwater	2	ND	-	19.606	44,250
Spiked groundwater	4	ND	-	17.662	173,072
Spiked groundwater	10	ND	-	16.556	375,871

(ND, not detected).

**Table 7:** Results of virus detection in eel farms (eel tissues and eDNA) using the duplex qPCR method developed in this study.

Farm	Samples	1st	2nd	3rd	4th	5th	6th
A (*A. japonica*)	Fin	ND	ND	JEECV 2 × 10^2^	JEECV 9 × 10	ND	ND
	Gill	ND	ND	JEECV 4 × 10^3^	JEECV 2 × 10^2^	JEECV 2 × 10	JEECV 3 × 10^0^
	Spleen	ND	ND	JEECV 1 × 10^2^	JEECV 6 × 10^0^	JEECV 2 × 10	JEECV 1 × 10
	Kidney	ND	ND	JEECV 6 × 10^2^	JEECV 2 × 10	JEECV 8 × 10^0^	JEECV 5 × 10^0^
	eDNA	ND	JEECV 1 × 10	JEECV 4 × 10	JEECV 1 × 10^2^	ND	ND
	Status	S	S	M	M	M	M
Detection of virus from fish							
				
Detection of virus from fish							
				
Fish mortalities							
				
Farm	Samples	1st	2nd	3rd	4th	5th	6th
B (*A. marmorata*)	Fin	ND	AnHV 1 × 10^4^	ND	AnHV 3 × 10^3^	ND	ND
	Gill	ND	AnHV 2 × 10^0^	ND	AnHV 4 × 10^5^	AnHV (D)	AnHV 4 × 10^5^
	Spleen	ND	AnHV 1 × 10^0^	ND	AnHV 1 × 10^5^	AnHV (D)	ND
	Kidney	ND	AnHV 4 × 10	ND	AnHV (D)	AnHV (D)	AnHV 2 × 10^5^
	eDNA	AnHV (D)	ND	ND	AnHV 1 × 10	ND	ND
	Status	S	S	W	M	M	M
Detection of virus from fish							
				
Detection of virus from eDNA							
					
Fish mortalities							
				

ND, not detected; D, detectable but not quantifiable due to trace-level detection (C_q_ value ≥ 35); S, stable; W, weakened; M, mortality.

## Data Availability

Data are contained within the article.

## Abbreviations

eDNA	environmental DNA
cPCR qPCR	conventional PCR quantitative PCR
JEECV	Japanese eel endothelial cells-infecting virus
AnHV	Anguillid herpesvirus 1
VECNE	eel viral endothelial cell necrosis
RASs	recirculating aquaculture systems
*Pol*	DNA polymerase catalytic subunit
*Ltlg*	polyomavirus large T-antigen-like protein gene
